# Tryptophan-Tyrosine Dipeptide, the Core Sequence of β-Lactolin, Improves Memory by Modulating the Dopamine System

**DOI:** 10.3390/nu11020348

**Published:** 2019-02-06

**Authors:** Yasuhisa Ano, Tatsuhiro Ayabe, Rena Ohya, Keiji Kondo, Shiho Kitaoka, Tomoyuki Furuyashiki

**Affiliations:** 1 Research Laboratories for Health Science & Food Technologies, Kirin Company Ltd, Kanazawa-ku, Yokohama-shi, Kanagawa 236-0004, Japan; Tatsuhiro_Ayabe@kirin.co.jp (T.A.); Rena_Ohya@kirin.co.jp (R.O.); kondok@kirin.co.jp (K.K.); 2 Division of Pharmacology, Kobe University Graduate School of Medicine, Kobe 650-0017, Japan; skitaoka@med.kobe-u.ac.jp (S.K.); tfuruya@med.kobe-u.ac.jp (T.F.); 3 AMED-CREST, Chiyoda-ku 100-0004, Tokyo, Japan

**Keywords:** dipeptide, dopamine, hippocampus, memory, monoamine oxidase B

## Abstract

Tryptophan-tyrosine (WY)-related peptides including the β-lactopeptide of the glycine-threonine-tryptophan-tyrosine peptide, β-lactolin, improve spatial memory. However, whether and how the WY dipeptide as the core sequence in WY-related peptides improves memory functions has not been investigated. This study assessed the pharmacological effects of the WY dipeptide on memory impairment to elucidate the mechanisms. Here, we showed that oral administration of dipeptides of WY, tryptophan-methionine (WM), tryptophan-valine, tryptophan-leucine, and tryptophan-phenylalanine improved spontaneous alternation of the Y-maze test in scopolamine-induced amnesic mice. In contrast, tyrosine-tryptophan, methionine-tryptophan, tryptophan, tyrosine, and methionine had no effect. These results indicated that the conformation of dipeptides with N-terminal tryptophan is required for their memory improving effects. WY dipeptide inhibited the monoamine oxidase B activity in vitro and increased dopamine levels in the hippocampus and frontal cortex, whereas tryptophan did not cause these effects. In addition, the treatment with SCH-23390, a dopamine D1-like receptor antagonist, and the knockdown of the hippocampal dopamine D1 receptor partially attenuated the memory improvement induced by the WY dipeptide. Importantly, WY dipeptide improved the spontaneous alternations of the Y-maze test in aged mice. These results suggest that the WY dipeptide restores memory impairments by augmenting dopaminergic activity. The development of supplements rich in these peptides might help to prevent age-related cognitive decline.

## 1. Introduction

The rapid growth of aging populations worldwide is associated with an increased incidence of cognitive decline and dementia that become a growing burden not only on patients and their families but also on national healthcare systems. Due to the lack of effective dementia therapies, increasing attention is given to preventive approaches. Recent epidemiological studies suggest that the consumption of certain dairy products reduces the risk of cognitive decline in the elderly and may prevent Alzheimer’s disease [1].

Crichton et al. [2] reported that individuals who consumed low-fat dairy products, including yogurt and cheese, once a week had a higher cognitive function than those who did not. A survey-based study of self-reported health information showed that the consumption of low-fat dairy products was associated with increased memory recall, increased social functioning, and decreased stress [3,4]. Ozawa et al. [3,4] surveyed more than 1000 Japanese subjects who were living in the community, aged 60–79 years, and free from dementia to investigate their dietary patterns and any potential association with a reduced risk of dementia symptoms. The authors concluded that including milk or fermented dairy products in the diet reduces the risk of dementia in the general Japanese population. In a clinical trial, Ogata et al. [5] investigated the association between the intake of dairy products and short-term memory and found that the intake of dairy products is highly associated with improved short-term memory. It has also been demonstrated that the intake of dairy products fermented with *Penicillium candidum*, i.e., Camembert cheese, had preventive effects on Alzheimer’s disease pathology in a mouse model [6].

We have previously identified tryptophan-tyrosine (WY)-related peptides, including the β-lactopeptide of glycine-threonine-tryptophan-tyrosine (GTWY) peptide, β-lactolin, derived from β-lactoglobulin in an enzymatic whey protein digest [7]. The β-lactolin was smoothly absorbed into the body and delivered to the brain where it was associated with a dopamine level increase, resulting in improved spatial and object memory [7]. In addition, we demonstrated that whey peptides rich in WY-related peptides improved memory and attention in a clinical trial [8]. These reports suggested that WY-related peptides improve cognitive function. However, whether and how the WY dipeptide, the core sequence of WY-related peptides, improves cognitive function has not been investigated. The gap in our knowledge about the pharmacological effects of the WY dipeptide on memory impairment hinders the assessment of the underlying mechanism of the effect of whey peptide preparations rich in WY-related peptides on cognitive performance in clinical trials.

In the present study, we examined the effects of tryptophan-containing dipeptides, including the WY dipeptide, on memory impairment and their mechanisms.

## 2. Materials and Methods

### 2.1. Materials

The dipeptides WY, tyrosine-tryptophan (YW), tryptophan-methionine (WM), methionine-tryptophan (MW), tryptophan-valine (WV), tryptophan-leucine (WL), and tryptophan-phenylalanine (WF) (purity > 98%) were purchased from NARD Chemicals, Ltd. (Amagasaki, Japan). Tryptophan, tyrosine, (−)-scopolamine hydrobromide trihydrate, and R(+)-SCH-23390 hydrochloride were purchased from Sigma Aldrich Co. (St. Louis, MO, USA).

### 2.2. Animals

Crl:CD1 (ICR) male mice, 6 weeks old, and C57BL/6J male mice, 7 months and 22 months old (Charles River Japan, Tokyo, Japan), were maintained at Kirin Company Ltd. Mice were maintained at room temperature (23 ± 1 °C) under a constant 12-h light/dark cycle (light period from 8:00 am to 8:00 pm) and were fed a standard rodent diet (CE-2 (Clea Japan, Tokyo, Japan)). Behavioral pharmacological tests were performed in a sound-isolated room with the same temperature and light/dark cycles. All efforts were made to minimize suffering. For euthanasia, mice were placed into a chamber filled with vapor of isoflurane (Wako, Osaka, Japan). All experiments were approved by the Animal Experiment Committee of Kirin Company Ltd and conducted in strict accordance with their guidelines in 2016.

### 2.3. Spontaneous Alteration Test

A spontaneous alternation test was conducted in accordance with our previous report [7]. The Y-maze is a three-arm maze with equal angles between all arms (25 cm long × 5 cm wide × 20 cm high). The maze walls were constructed from dark black, polyvinyl plastic. Each mouse was initially placed in one arm, and the sequence and number of arm entries were counted for 8 minutes. The alternation score (%) for each mouse was defined as the ratio of the actual number of alternations to the possible number (defined as the total number of arm entries minus two) multiplied by 100 as follows: % Alternation = ((Number of alternations) / (Total arm entries − 2)) × 100.

To evaluate the effects of the samples on scopolamine-induced memory impairment, 6-week-old Crl:CD1 male mice were orally administered distilled water with or without peptides to be tested 1 h before evaluation. Forty minutes after the oral administration, memory impairment was induced by the intraperitoneal administration of 0.85 mg/kg scopolamine dissolved in saline. One hour after the oral administration, mice were subjected to the Y-maze test. In some experiments, mice were administered 0.85 mg/kg scopolamine and 0.05 mg/kg SCH-23390 (dopamine D1 antagonist) intraperitoneally at 40 min after the oral administration of 0 or 1 mg/kg WY peptide and subjected to the Y-maze test at 1 h after the oral administration. There were 10 mice per group.

In other experiments, C57BL/6J mice were orally administered a daily dose of 0 or 1 mg/kg WY peptide for 14 days. One hour after the last administration, mice were subjected to a Y-maze trial.

### 2.4. Monoamine Analysis

To evaluate the levels of dopamine and its metabolites (DOPAC and HVA) in the brain, tissue was homogenized in 0.2 M perchloric acid (PCA, Wako, Tokyo, Japan) containing 100 μM EDTA·2Na (Sigma-Aldrich, St. Louis, MO, USA). After centrifugation, the supernatant was analyzed by high-performance liquid chromatography (HPLC) using an EICOMPAK SC-5ODS column and PREPAK column (Eicom, Kyoto, Japan) with an electrochemical detection (ECD) unit. The mobile phase consisted of 83% 0.1 M acetic acid in citric acid buffer (pH 3.5), 17% methanol (Wako), 190 mg/ml of sodium 1-octanesulfonate sodium (Wako), and 5 mg/mL EDTA·2Na. For ECD, the applied voltage was 750 mV vs. an Ag/AgCl reference electrode.

### 2.5. Monoamine Oxidase (MAO) Activity Assay

MAO-B activity was measured using the MAO assay kit (Cell Biolabs, San Diego, CA, USA) according to the manufacturer’s instructions. Human MAO-B (50 μg/mL; Sigma-Aldrich) was incubated with 1 mM WY dipeptide or 1 mM tryptophan for 30 min. The substrates for MAO-B were added and the amount of hydrogen peroxide in the reaction was measured.

### 2.6. Injection of Adeno-Associated Viruses (AAV) to the Hippocampus

The adeno-associated virus (AAV) injection method used to knock down the dopamine D1 receptor and the following behavioral evaluation were performed as described in our previous study [9,10]. The AAV construct that expresses artificial microRNA (miRNA) targeting the dopamine D1 receptor with an emerald green fluorescent protein (EmGFP) under the control of the elongation factor (EF)1α promoter only in the presence of Cre recombinase (AAV10-EF1α-double-floxed inverted (DIO)-EmGFP-D1miRNA), an AAV construct that expresses control miRNA in the same arrangement (AAV10-EF1α-DIO-EmGFP-control), and an AAV construct that expresses Cre recombinase under the CMV promoter (AAV10-CMV-Cre) were produced as previously described [9]. After anesthetized with sodium pentobarbital, 8-week-old Crl:CD1 male mice were injected with 0.5 μL of the AAV solution (1.0 × 10^12^ genomics copies/mL) per site, applied at two sites in the hippocampal regions of both hemispheres (four sites per mouse; from bregma: posterior, −3.5 mm; lateral, ± 3 mm; ventral, −3.8 mm and −1.8 mm) according to the atlas of Paxinos and Franklin [11] and using a PV-830 Pneumatic PicoPump (World Precision Instruments, Sarasota, FL, USA). Mice were allowed to recover for 4 weeks and then used for the Y-maze test.

### 2.7. Statistical Analysis

All values are expressed as means ± standard error of the mean (SEM). Data was analyzed by one-way analysis of variance (ANOVA), followed by Tukey–Kramer test, Dunnett’s test, or Student’s *t*-test as described in the figure legends. All statistical analyses were performed using the Ekuseru–Toukei 2012 software program (Social Survey Research Information, Tokyo, Japan). A *p*-value < 0.05 was considered statistically significant.

## 3. Results

### 3.1. Tryptophan-Containing Dipeptides Improved Memory Impairment in Amnesic Mice

**Figure 1:**
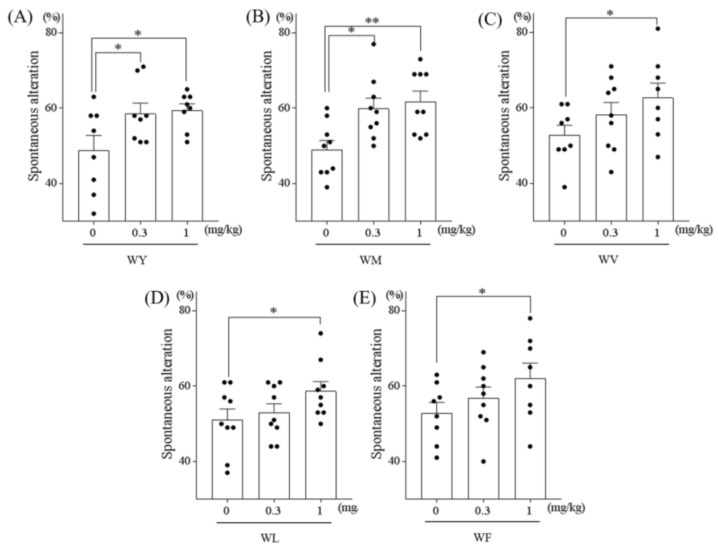
The effects of dipeptides (**A**) WY, (**B**) WM, (**C**) WV, (**D**) WL, and (**E**) WF on spatial memory in amnesic mice. Six-week-old Crl:CD1 male mice were orally administered 0, 0.3, or 1 mg/kg of dipeptide (WV, WM, WV, WL, and WF) and, 40 min later, injected intraperitoneally with 0.85 mg/kg of scopolamine. At 1 h after oral administration, each mouse was allowed to explore the Y-maze for 8 min. Spontaneous alternations were also measured. Data represent the mean ± SEM of 10 mice per group. The *p* values shown were calculated using the Dunnett’s test. *p < 0.05 and **p < 0.01.

To evaluate the effects of the WY, WM, WV, WL, and WF dipeptides, which are known to be derived from milk proteins, on spatial memory, amnesia was induced by treatment with scopolamine, a muscarinic antagonist, according to a previous study [7]. The spontaneous alternation test using the Y-maze is well-established as a behavioral evaluation method for examining short-term spatial memory performance. A single dose of 1 mg/kg WY, WM, WV, WL, or WF dipeptide significantly increased the spontaneous alternation (Figure 1A–E, respectively). Further, the administration of 0.3 mg/kg WY or WM dipeptide already increased the alternation, which showed higher improvement than that of WV, WL, or WF (Figure 1A and B). The number of arm entries was not changed among the groups (data not shown). These results indicated that the administration of certain dipeptides with N-terminal tryptophan improved short-term spatial memory in amnestic mice.

### 3.2. Dipeptides Containing Tryptophan at the N-Terminus But Not at the C-Terminus Improved Memory Impairment

**Figure 2:**
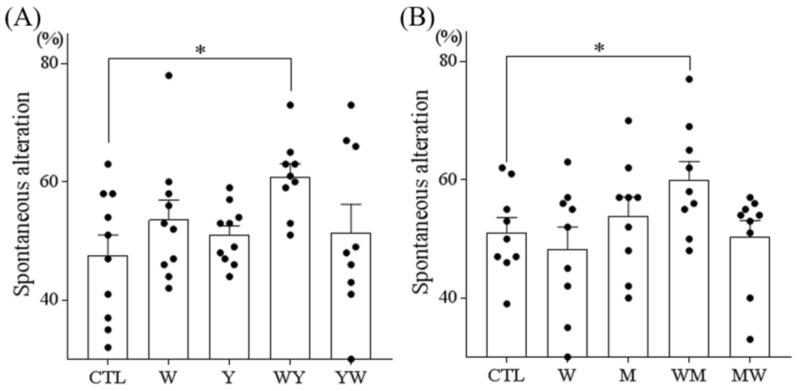
The effects of the dipeptides and single amino acids of (A) WY and (B) WM on spatial memory in amnesic mice. Six-week-old Crl:CD1 male mice were orally administered 0 or 1 mg/kg of dipeptide or single amino acid (WY, YW, WM, MW, tryptophan (W), tyrosine (Y), and methionine (M)) and, 40 min later, injected intraperitoneally with 0.85 mg/kg of scopolamine. At 1 h after oral administration, each mouse was allowed to explore the Y-maze for 8 min. Spontaneous alternations were also measured. Data represent the mean ± SEM of 10 mice per group. The *p* values shown were calculated using the Dunnett’s test. * *p* < 0.05.

Next, to evaluate the effect of the tryptophan position within the dipeptides, we assessed the effect of tryptophan, tyrosine, and the dipeptides WY and YW on spatial memory in the spontaneous alternation test. A single administration of 1 mg/kg WY dipeptide, but not tryptophan, tyrosine, or YW dipeptide, increased the spontaneous alternation (Figure 2A). We also tested the effect of tryptophan, methionine, and the dipeptides WM and MW on spatial memory. A single administration of 1 mg/kg WM peptide, but not tryptophan, methionine, or MW dipeptide, also increased the alternation (Figure 2B). These results suggested that the conformation of dipeptides with an N-terminal tryptophan is required to improve the spatial memory in amnestic mice.

### 3.3. WY Peptide Increased Dopamine Levels in the Hippocampus and Frontal Cortex

**Figure 3:**
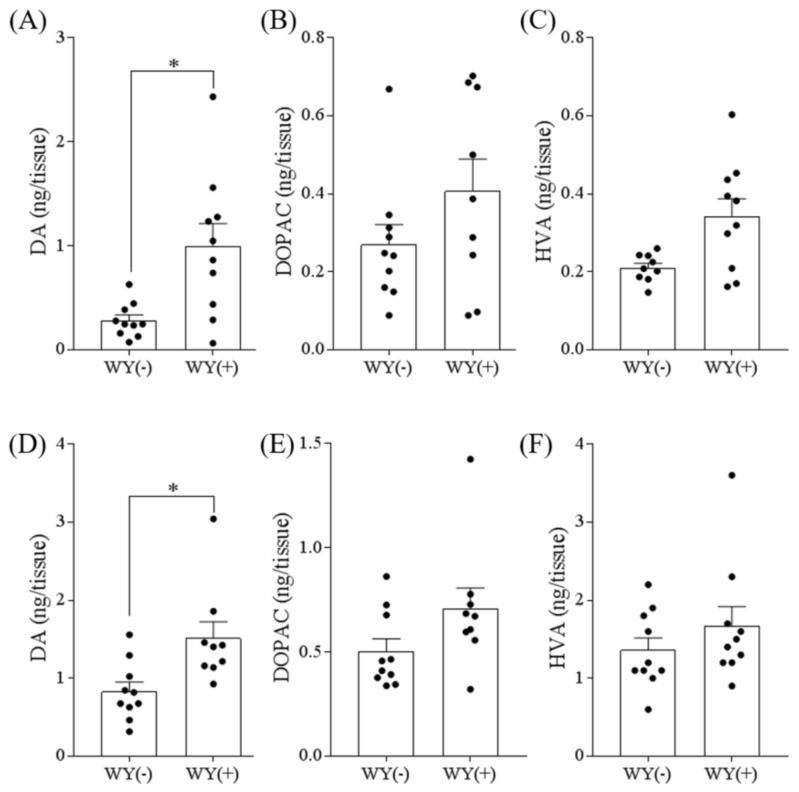
The levels of dopamine and its metabolites in the hippocampus and frontal cortex. Six-week-old Crl:CD1 male mice were orally administered 0 or 1 mg/kg of WY dipeptide. At 1 h after oral administration, the following monoamine levels were measured in the hippocampus (**A**–**C**) and frontal cortex (**D**–**F**) by HPLC: dopamine (DA) (**A**, **D**), 3,4-dihydroxyphenylacetic acid (DOPAC) (**B**, **E**), and homovanillic acid (HVA) (**C**, **F**). Data represent the mean ± SEM of 10 mice per group. The *p* values shown were calculated using the Student’s *t*-test. * *p* < 0.05.

Because we previously reported that the GTWY peptide inhibits MAO-B activity in vitro and in vivo and increases dopamine contents in the frontal cortex and hippocampus, we further evaluated the effect of the WY dipeptide on the catecholamine levels in the hippocampus and frontal cortex. In both the hippocampus and frontal cortex, a single administration of the WY dipeptide significantly increased the level of dopamine (Figure 3A–F). The levels of DOPAC and HVA appear to be slightly increased, though not statistically significant. Thus, the administration of WY dipeptide increased the level of dopamine in the brain without affecting the levels of its metabolites.

### 3.4. WY Peptide Inhibited the Activity of MAO

**Figure 4:**
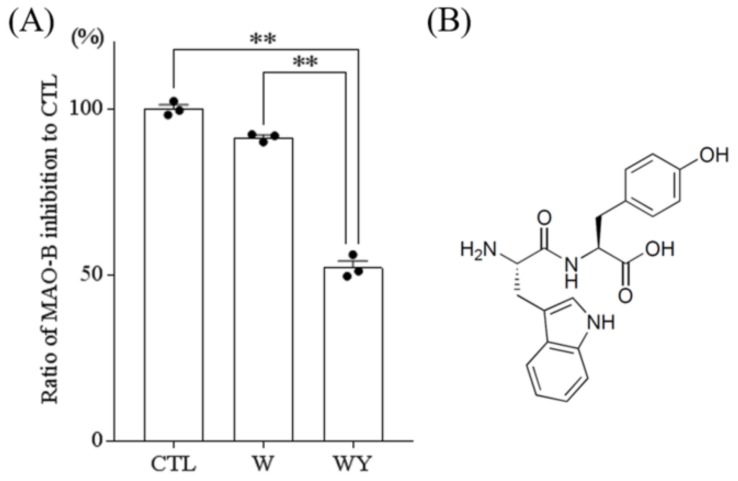
The inhibition of monoamine oxidase by the WY dipeptide and tryptophan and the chemical structure of the WY dipeptide. (**A**) The activity of monoamine oxidase B (MAO-B) was tested in the presence of 1 mM WY dipeptide or tryptophan (W). Data represent the mean ± SEM of triplicate per sample. The *p* values shown were calculated using the Dunnett’s test. ** *p* < 0.01. (**B**) The chemical structure of the WY dipeptide.

We evaluated the effect of WY dipeptide and tryptophan on MAO-B activity. Tyrosine and YW dipeptide were not tested in this assay because the compounds could not be dissolved in the assay buffer. Treatment with 1 mM WY dipeptide decreased MAO-B activity by 48 ± 1.95% compared to that of the control treatment. By contrast, treatment with 1 mM tryptophan did not inhibit MAO-B activity (Figure 4A). These results suggested that the WY dipeptide increased the dopamine content in the hippocampus by inhibiting MAO-B activity and that its chemical structure (Figure 4B) was important to inhibit the MAO-B activity.

### 3.5. Inhibition of the Dopamine D1 Receptor Attenuated the WY-Induced Memory Improvement

**Figure 5:**
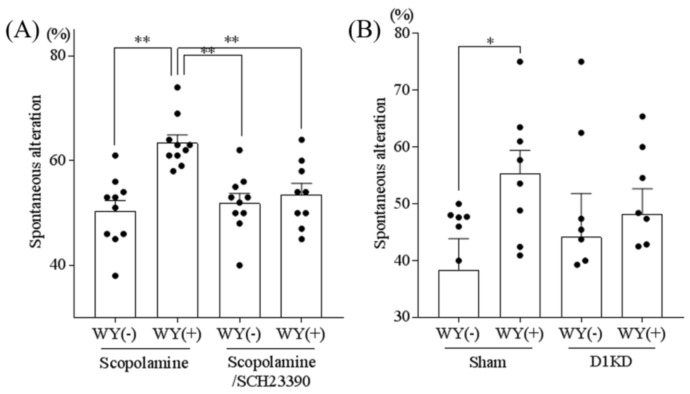
The dopamine receptor is involved in memory improvement linked to the WY dipeptide. (**A**) Six-week-old Crl:CD1 male mice were orally administered 0 or 1 mg/kg of WY dipeptide and, 40 min later, intraperitoneally injected with 0.85 mg/kg of scopolamine alone or scopolamine plus 0.05 mg/kg of SCH-23390. At 1 h after oral administration of the peptide, mice were allowed to explore the Y-maze for 8 min. Data represent the mean ± SEM of 10 mice per group. (**B**) Eight-week-old Crl:CD1 male mice were administered either a control microRNA or dopamine D_1_ receptor microRNA containing AAV, which suppresses the dopamine D_1_ receptor, to the hippocampus. Mice were orally administered 0 or 1 mg/kg of WY dipeptide and, 40 min later, intraperitoneally injected with 0.85 mg/kg of scopolamine. At 1 h after oral administration of the peptide, mice were allowed to explore the Y-maze for 8 min. Data represent the mean ± SEM of 7–8 mice per group. The *p* values were calculated using one-way ANOVA followed by the Tukey–Kramer test. ** *p* < 0.01 and * *p* < 0.05.

To investigate the link between dopamine and the memory-improving effect of the WY dipeptide, we examined the effect of SCH-23390, a dopamine D1-like receptor antagonist on the WY-dipeptide-induced memory improvement. Whereas WY-dipeptide increased spontaneous alteration in scopolamine-induced amnestic mice with prior treatment with saline (control group), SCH-23390 treatment abolished the memory improvement induced by the WY dipeptide (Figure 5A). These results indicated that the dopamine D1-like receptor was involved in the improvement of spatial memory induced by the WY dipeptide.

To further determine the involvement of the dopamine D1 receptor in the memory-improving effect of the WY dipeptide, the mRNA expression of the dopamine D1 receptor was knocked down in hippocampal neurons using the AAV system expressing artificial miRNA targeting this receptor according to our previous study [10]. While the WY dipeptide administration increased the spontaneous alternation in amnestic mice expressing control miRNA, this memory improvement was not significant with the dopamine D1 receptor knockdown in hippocampal neurons (Figure 5B). These results suggested that the dopamine D1 receptor in the hippocampus is involved in the memory improvement caused by the WY dipeptide at least in part.

### 3.6. WY Peptide Improved Age-Related Memory Impairment

**Figure 6:**
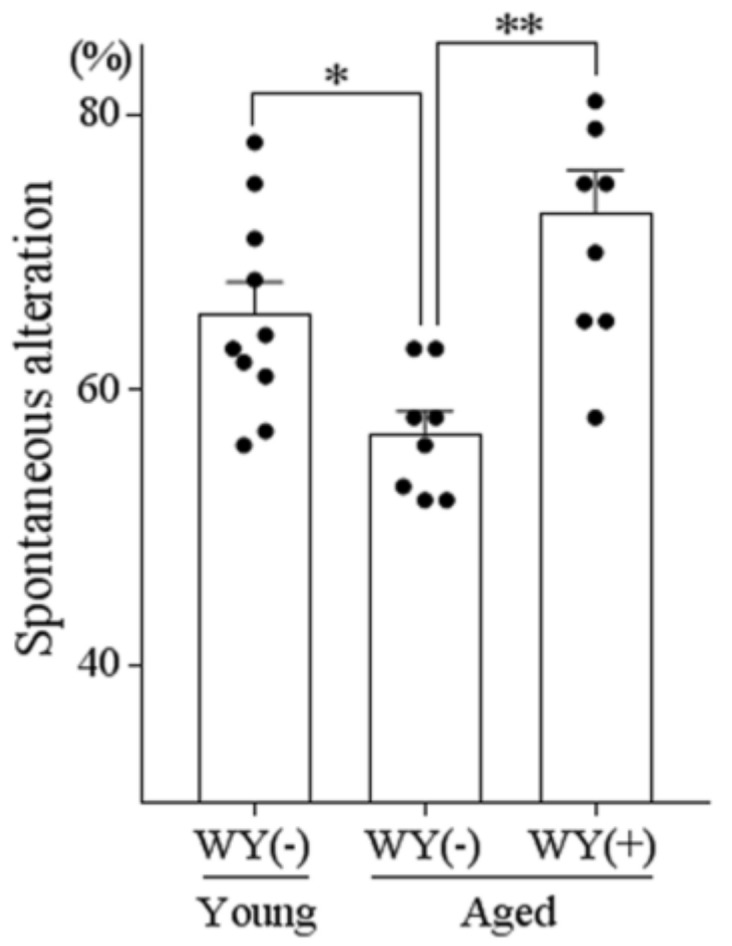
The effects of the WY peptide on aged mice: 7- and 22-month-old C57BL/6 mice were orally administered a daily dose 1 mg/kg of WY dipeptide for 14 days. At 1 h after oral administration of the peptide, mice were allowed to explore the Y-maze for 8 min. Data represent the mean ± SEM of 8–10 mice per group. The *p* values were calculated using one-way ANOVA followed by the Tukey–Kramer test. * *p* < 0.05 and ** *p* < 0.01.

Our results showed that the WY dipeptide restored pharmacologically-induced memory impairment in mice. Therefore, we further examined whether the WY dipeptide would have a similar effect on memory impairment in aged mice. Aged (22 months) and young (7 months) C57BL/6J mice were orally administered WY dipeptide, and their performance in the spontaneous alternation test was evaluated. The proportion of spontaneous alternation was reduced in aged mice, compared with young mice, indicating age-related memory impairment. The administration of the WY dipeptide increased the proportion of spontaneous alteration in aged mice (Figure 6), indicating that WY dipeptide also restored memory impairment in aged mice.

## 4. Discussion

Epidemiological studies have reported that the consumption of fermented dairy products is beneficial for the prevention of cognitive decline in the elderly [1]. Our previous study demonstrated that WY-related peptides derived from enzymatic whey protein digests, such as the GTWY peptide β-lactolin, improve memory impairment in mice with pharmacologically-induced amnesia [7]. However, the underlying mechanism of the effects of the WY dipeptide core included in WY-related peptides on memory improvement is not well-understood. In the present study, we demonstrated that dipeptides with an N-terminal tryptophan, such as WY, WM, WV, WL, and WF improved memory impairment in scopolamine-induced amnesic mice. Especially, the WY dipeptide increased the dopamine levels in the hippocampus and frontal cortex, and the WY-induced memory improvement was attenuated by the blockade of the dopamine D1 receptor. Moreover, the WY dipeptide improved spatial memory impairment in aged mice. These results suggested that orally administered WY dipeptide improved spatial memory impairments in pharmacologically-induced amnestic mice and aged mice via modulating the dopamine system.

We showed that the WY dipeptide inhibited MAO-B activity and increased dopamine levels in the hippocampus and frontal cortex and improved spatial memory impairment in pharmacologically-induced amnestic mice and aged mice. Previous reports have indicated that dopamine is involved in hippocampus-dependent memory functions [12,13]. The dopamine neuronal network is related to an age-related decline in cognitive performance and executive function, and the dopamine precursor levodopa has been shown to improve the task-based learning rate and task performance in the elderly [14]. It has also been reported that MAO-B inhibitors improve cognitive function in rodents [15,16,17]. MAO-B is an enzyme for dopamine metabolism and thus decreases dopamine levels in the brain [18]. Therefore, MAO-B inhibitors have been used as drugs to induce dopamine levels [19]. It is suggested that some MAO-B inhibitors have a potential as therapeutic or preventive treatment for dementia including Alzheimer’s disease [20,21]. We also showed that the administration of WY dipeptide increased total dopamine levels in the hippocampus and frontal cortex. These results suggest that WY dipeptide increase dopamine levels in the brain by inhibiting MAO-B activity. Those findings suggest that the WY dipeptide exerts its beneficial effect on the prevention of age-related cognitive decline by increasing dopaminergic activity in the brain.

In addition, we showed that dopamine D1 receptor is involved in the spatial memory improvement induced by the WY dipeptide. Dopamine exerts its functions via multiple receptor subtypes, D1-like (D1 and D5) and D2-like (D2, D3, and D4) receptors. In the present study, the treatment with D1-like receptor antagonist, SCH-23390 attenuated the improvement caused by the WY dipeptide in scopolamine-induced amnesic mice. It has been reported that activation of dopamine D1-like receptors enhances hippocampus-dependent memory functions [22,23,24,25]. Because the hippocampus is crucial for spatial memory as measured by the Y-maze test [26], we examined the involvement of the dopamine D1 receptor in the hippocampus in WY-dipeptide-induced memory improvement. Dopamine D1-like receptors are expressed in many brain areas including the hippocampus, but due to the lack of selective pharmacological drugs, most studies failed to discriminate dopamine receptor subtypes. Recently, the brain-region-specific knockdown of the dopamine D1 receptor subtype was achieved by injecting AAV vectors that express recombinant miRNA targeting this dopamine receptor subtype [9]. This technique made it possible to further examine a potential link between the spatial memory improvement by the WY dipeptide and the dopamine D1 receptors in the hippocampus. Dopamine D1 receptor knockdown in the hippocampus attenuated the improvement by the WY peptide, but it was a partial reduction. These data suggested that the dopamine D1 receptor in the hippocampus is involved in the WY-dipeptide-induced memory improvement for spatial information measured in the Y-maze test at least in part. The reason why the effect of the D1 receptor knockdown was partial remains unclear. It could be due to the partial knockdown of the dopamine D1 receptor in the hippocampus. This finding is consistent with previous reports suggesting that D1 receptor stimulation in the hippocampus augments spatial memory. Alternatively, dopamine in the frontal cortex could be involved in the WY-dipeptide-induced memory improvement, since the WY dipeptide increased the dopamine levels in frontal cortex as well.

In the present study, we compared the effects of several dipeptides containing tryptophan and tryptophan as a single amino acid. We found that the administration of a single dose of dipeptides with an N-terminal tryptophan, WY, WM, WV, WL, and WF improved spatial memory in mice, whereas YW and MW dipeptides did not have this effect. These findings suggest that the dipeptide conformation with an N-terminal tryptophan is crucial for effectively improving the memory impairment after oral administration. It has been shown that dipeptides with an N-terminal tryptophan have a high affinity to peptide transporters, and among the dipeptide library, the WY dipeptide is one of the dipeptides with the highest affinities [27]. Thus, the conformation of dipeptides with an N-terminal tryptophan could be necessary for transporter-mediated absorption from the gut and delivery to the brain. On the other hand, based on our finding that the WY dipeptide, but not tryptophan as a single amino acid, inhibited MAO-B activity in vitro, the dipeptide conformation with an N-terminal tryptophan could be crucial for enhancing dopaminergic activity. Note that these two possibilities are not mutually exclusive but rather synergetic for the memory improving effect of the WY dipeptide.

## 5. Conclusions

In summary, the present study showed that the WY dipeptide improves pharmacologically-induced and age-related memory impairment in mice. Effective intake of the WY dipeptide and other dipeptides with N-terminal tryptophan via supplements or nutraceutical foods including certain whey peptide preparations might be beneficial for improving cognitive function in older age.
